# Immunization with GP1 but Not Core-like Particles Displaying Isolated Receptor-Binding Epitopes Elicits Virus-Neutralizing Antibodies against Junín Virus

**DOI:** 10.3390/vaccines10020173

**Published:** 2022-01-22

**Authors:** Gleyder Roman-Sosa, Anne Leske, Xenia Ficht, Tung Huy Dau, Julia Holzerland, Thomas Hoenen, Martin Beer, Robert Kammerer, Reinhold Schirmbeck, Felix A. Rey, Sandra M. Cordo, Allison Groseth

**Affiliations:** 1 Department of Internal Medicine I, Ulm University Hospital, 89081 Ulm, Germany; ficht.xenia@hsr.it (X.F.); reinhold.schirmbeck@uni-ulm.de (R.S.); 2 Laboratory for Arenavirus Biology, Institute of Molecular Virology and Cell Biology, Friedrich-Loeffler-Institut, 17493 Greifswald, Germany; anne.leske@fli.de (A.L.); julia.holzerland@fli.de (J.H.); 3 Laboratory for Immunogenetics, Institute of Immunology, Friedrich-Loeffler-Institut, 17493 Greifswald, Germany; tunghuydau@gmail.com (T.H.D.); robert.kammerer@fli.de (R.K.); 4 Laboratory for Integrative Cell and Infection Biology, Institute of Molecular Virology and Cell Biology, Friedrich-Loeffler-Institut, 17493 Greifswald, Germany; thomas.hoenen@fli.de; 5 National and OIE Reference Laboratory for BHV-1, Institute of Diagnostic Virology, Friedrich-Loeffler-Institut, 17493 Greifswald, Germany; martin.beer@fli.de; 6 Structural Virology Unit, CNRS UMR3569, Institut Pasteur, Université de Paris, 75015 Paris, France; rey@pasteur.fr; 7 Instituto de Química Biológica de la Facultad de Ciencias Exactas y Naturales (IQUIBICEN), University of Buenos Aires, Ciudad Universitaria, Pabellón II, Piso 4, Buenos Aires 1428, Argentina; scordo@qb.fcen.uba.ar

**Keywords:** arenavirus, Junín virus, immune response, neutralizing antibodies, glycoprotein, GP1

## Abstract

New World arenaviruses are rodent-transmitted viruses and include a number of pathogens that are responsible for causing severe human disease. This includes Junín virus (JUNV), which is the causative agent of Argentine hemorrhagic fever. The wild nature and mobility of the rodent reservoir host makes it difficult to control the disease, and currently passive immunization with high-titer neutralizing antibody-containing plasma from convalescent patients is the only specific therapy. However, dwindling supplies of naturally available convalescent plasma, and challenges in developing similar resources for other closely related viruses, have made the development of alternative antibody-based therapeutic approaches of critical importance. In this study, we sought to induce a neutralizing antibody response in rabbits against the receptor-binding subunit of the viral glycoprotein, GP1, and the specific peptide sequences in GP1 involved in cellular receptor contacts. While these specific receptor-interacting peptides did not efficiently induce the production of neutralizing antibodies when delivered as a particulate antigen (as part of hepatitis B virus core-like particles), we showed that recombinant JUNV GP1 purified from transfected mammalian cells induced virus-neutralizing antibodies at high titers in rabbits. Further, neutralization was observed across a range of unrelated JUNV strains, a feature that is critical for effectiveness in the field. These results underscore the potential of GP1 alone to induce a potent neutralizing antibody response and highlight the importance of epitope presentation. In addition, effective virus neutralization by rabbit antibodies supports the potential applicability of this species for the future development of immunotherapeutics (e.g., based on humanized monoclonal antibodies). Such information can be applied in the design of vaccines and immunogens for both prevention and specific therapies against this and likely also other closely related pathogenic New World arenaviruses.

## 1. Introduction

Arenaviruses include a number of rodent-borne pathogens that can infect mammals. They are divided into those found in Eurasia and Africa (i.e., Old World (OW) arenaviruses) [1] and the Americas (i.e., New World (NW) arenaviruses) [2]. The latter include several important agents of human disease, including Guanarito virus, Sabiá virus, Chapare virus, Machupo virus (MACV), and Junín virus (JUNV), all of which cause hemorrhagic fever [3,4]. To date, JUNV, which causes Argentine hemorrhagic fever (AHF), has been the most medically significant of the NW arenaviruses, having caused tens of thousands of infections since its discovery in the 1950s. In nature, JUNV circulates in its reservoir, the drylands vesper mouse (*Calomys musculinus*), with transmission to humans occurring through direct contact with infected rodents or inhalation of infectious aerosols from rodent excretions/secretions [5]. Disease is characterized by early non-specific febrile symptoms that develop in 20–30% of cases to include hemorrhages and/or neurological symptoms, which are indicative of a poor prognosis [6] and contribute to the 15% to 20% case fatality rate associated with untreated AHF.

A major factor in the control of AHF has been the widespread use of a live-attenuated vaccine (Candid#1) in high-risk populations [6]; however, supplies are limited. Additionally, there is a specific therapy available, which relies on plasma from convalescent patients, but limited availability and variability in neutralizing antibody titers among donors pose challenges to its use and the long-term sustainability of this approach. Similar resources for other related NW arenaviruses are currently unavailable. Hence, alternative approaches to vaccine design and new sources of immune therapeutics are still critically needed to support control efforts against these viruses. Given the success of passive immunization in controlling infection, the induction of antibodies that block viral entry appears to be a promising strategy for their development.

JUNV has a bipartite single-stranded RNA genome that encodes the RNA-dependent RNA polymerase (L) and matrix protein (Z) on the L segment, and the nucleocapsid protein (NP) and the envelope glycoprotein on the S segment. The glycoprotein is translated as a precursor (GPC) before being cleaved by subtilisin kexin isozyme-1/site-1 protease (SKI-1/S1P) into GP1 and GP2 as it matures through the endoplasmic reticulum/Golgi network [7,8]. The mature GP1 and GP2 subunits associate as trimers of heterodimers on virus particles. The attachment of virions to the target cell occurs via the receptor-binding subunit GP1, while GP2 is responsible for fusion of the viral and target cell membranes.

**Figure 1:**
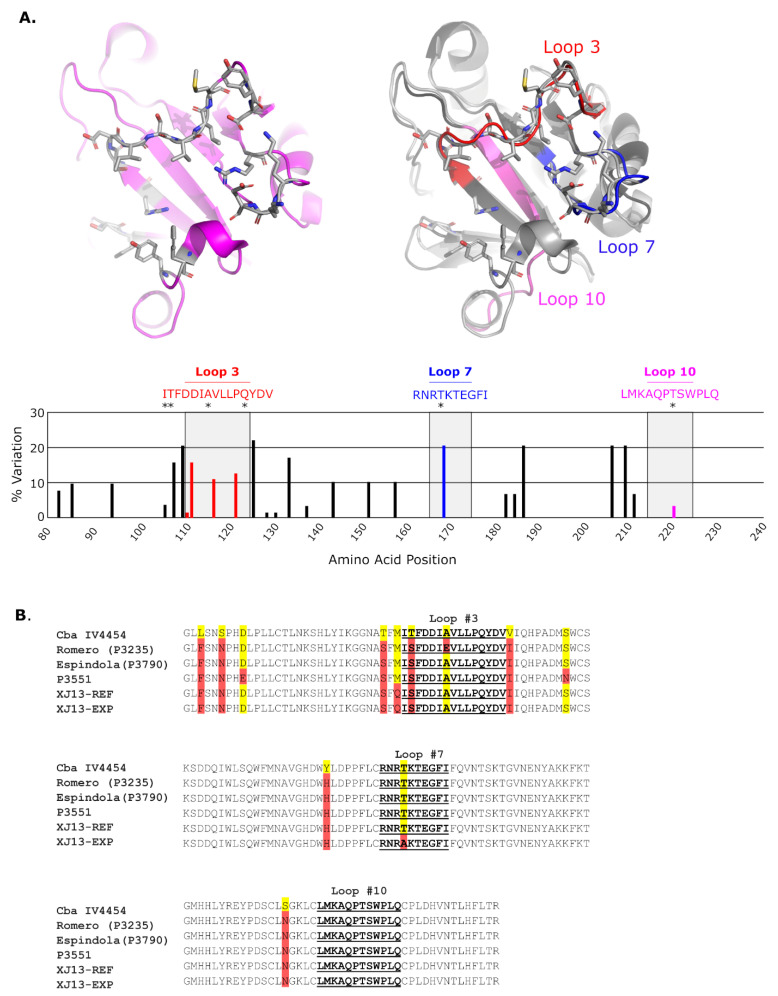
Strategy for the generation of Junín virus (JUNV)-derived peptide antigens for expression on hepatitis B virus core-like particles. (**A**) Crystal structures of Machupo virus (MACV) GP1 and JUNV GP1. The X-ray structure of MACV GP1 in complex with transferrin receptor 1 (TfR1) (PDB:3KAS [11]), in which the TfR1 atoms were removed, is represented in the left panel. GP1 is colored pink except for the residues making contact with TfR1 in the complex, which are shown as sticks (color coded according to the atom type: carbon, light grey; nitrogen, blue; oxygen, red; sulfur, yellow). The right panel shows an overlay of the same structure with JUNV GP1 (extracted from the X-ray structure of JUNV GP1 in complex with Fab GD01, PDB:5EN2 [12]). The MACV GP1 structure is represented with its interacting residues as sticks, as in the left panel, except that the rest of the protein is in grey. The JUNV GP1 structure is shown only as ribbons in which the three loops tested are colored, as indicated. Note that Loop 3 extends all along the interaction surface and could constitute a potential linear epitope eliciting neutralizing antibodies. These two panels were prepared with the PyMOL Molecular Graphics System (Version 2.1, Schrödinger). Conservation of the amino acids in and around the respective loops (amino acid positions 80–240) are shown based on an alignment of the sequences of 63 unique naturally occurring JUNV isolates available in GenBank (Figure S2). The positions of the respective loops are boxed and bars indicating the frequency of variable amino acids within these regions are shown in color. The corresponding peptide sequences are shown, and asterisks indicate these variable sites. (**B**) Sequence alignment for JUNV strains used in this study. Details of the sequence alignment are shown for the region from amino acid 80 to 240 for the CbaIV4454 strain (GenBank: DQ272266), against which the peptide sequences were designed, and the other strains used for the testing of cross-neutralization in this study. Sequences corresponding to the selected peptide loops are indicated in bold text and underlined. Sequences for strains Romero (GenBank: JN801476), Espindola (GenBank: DQ854739), and P3551 (GenBank: MZ408913, this study), and both a reference sequence for strain XJ13 (XJ13-REF; GenBank: FJ805378) and the sequence determined based on the XJ13 isolate available in our laboratory (XJ13-EXP; GenBank: MZ408914) are shown. Sites showing amino acid variation are highlighted in color, with the amino acid observed in strain CbaIV4454 shown in yellow and all other variants shown in red. *: “asterisks indicate these variable sites“.

Transferrin receptor (TfR1) plays a key role as a receptor, and the use of the human orthologue is a common feature of highly pathogenic NW arenaviruses [9,10]. Structural analysis of the MACV GP1-TfR1 complex [11] has revealed the domains involved in this interaction. Further, comparison with the structure of JUNV GP1 suggests that the interaction interface on GP1, which consists of three protruding loops that face and make contact with TfR1 (highlighted in Figure 1), is highly conserved [12]. Consistent with these structural data, an antibody that binds to this domain in human TfR1 (hTfR1) effectively blocks the entry of NW arenaviruses in vitro [9], highlighting the GP1–receptor interaction as a promising target for the development of therapeutic antibodies. Further, the identification of the antigens/epitopes that induce pathogen-neutralizing antibodies can also facilitate the design of vaccine candidates. For instance, the receptor-binding domain of SARS-CoV2, which is targeted by neutralizing antibodies [13], also provides protection in an animal model when used alone as an immunogen [14]. Therefore, based on the available structural information for JUNV GP1, including the GP1–TfR1 interface, we considered whether this could be used to support a structure-assisted approach to optimizing the humoral immune response to the glycoprotein by focusing it towards structural determinants that are crucial for receptor recognition. To examine the potential of such an approach, the present study aimed to induce virus-neutralizing antibodies based on immunization with either recombinant JUNV GP1 or specific GP1-derived epitopes that interact with hTfR1 presented at an immunogenic position in a particulate antigen. However, while we could show that immunization with soluble JUNV GP1 indeed generates a robust neutralizing antibody response in immunized rabbits, the receptor-interacting peptides were not immunogenic when expressed in isolation even when delivered as part of a highly immunogenic particulate antigen.

## 2. Materials and Methods

### 2.1. Generation of DNA Constructs for Protein Expression

Plasmids for recombinant expression of the JUNV (strain CbaIV4454) GP1 were generated by PCR amplification of the sequence corresponding to amino acids 86 to 246 from a plasmid encoding the entire GPC polyprotein (GenBank Accession: DQ272266) and cloning into the pHL-Sec vector (kindly provided by Yvonne Jones, University of Oxford) [15] using standard molecular biology methods. Two versions of the construct were prepared, containing either a Strep tag (pHL-Sec-JUNV GP1-Strep) or a 6 × Histidine tag (pHL-Sec-JUNV GP1-His_6_) tag at the carboxyl terminus.

Plasmids for hepatitis B virus core-like particle (HBV CLP) generation were based on constructs containing the full-length HBV core protein (amino acids 1–183) with an amino terminal twin Strep tag in the vector pET-28a(+) (Novagen, Darmstadt, Germany). Further, this construct contained a FLAG tag, flanked by serine-glycine linkers at the immunodominant c/e1 epitope (pET28a-HBVc-FLAG) [16]. Sequences encoding JUNV Loop 3 (pET28a-HBVc-JUNV Loop 3), Loop 7 (pET28a-HBVc-JUNV Loop 7), or Loop 10 (pET28a-HBVc-JUNV Loop 10) were exchanged against the FLAG tag, to generate constructs that instead expressed these virus-derived peptides at the immunodominant c/e1 epitope. All constructs (Supplementary Materials Text S1) were generated using standard molecular biology methods and further details of the cloning strategies can be provided upon request.

### 2.2. Expression and Purification of Strep-Tagged JUNV GP1

Recombinant Strep-tagged JUNV GP1 was isolated from the supernatant of transiently transfected HEK-293 cells as previously described [17]. Briefly, the pHL-Sec-JUNV GP1-Strep was complexed with branched polyethylenimine (PEI; Sigma-Aldrich, Burlington, MA, USA) at a PEI:DNA ratio of 3:1 in serum-free medium. The mixture was incubated at room temperature for 15 min and was added to the cells. After 3 h of incubation, the transfection reagent was removed and replaced by complete medium. At 3 days post-transfection, the supernatant was collected, clarified of cell debris by low-speed centrifugation (10 min, 500× *g*), and concentrated by tangential flow filtration at a flow rate of 10 mL/min using a Vivaflow cassette with a molecular weight cut-off (MWCO) of 5000 Da (Sartorius Stedim Biotech, Göttingen, Germany). The protein was then purified by affinity chromatography using Strep-Tactin Super-flow high-capacity slurry (IBA Lifesciences, Göttingen, Germany) following the manufacturer’s instructions. The obtained elution fractions were analyzed by SDS-PAGE and the proteins were stained with Instant blue (Expedeon, Cambridge, UK). Fractions containing the protein were pooled and concentrated through an Amicon Ultra filter with an MWCO of 3000 Da (Millipore, MA, USA) at 5000× *g* for 20–30 min. The protein concentration was determined using the Bradford method (Quick Start Bradford Dye Reagent; Bio-Rad, Hercules, CA, USA). The final protein preparation was stored at −80 °C until use.

### 2.3. Expression and Purification of Histidine-Tagged JUNV GP1

FreeStyle 293-F cells were resuspended in fresh FreeStyle 293 Expression Medium (FEM) (Life Technologies, Carlsbad, CA, USA) at 3 × 10^6^ cells/mL and transiently transfected with pHL-Sec-JUNV GP1-His_6_. The DNA was diluted in FEM to 0.5 μg/μL, vortexed, and briefly centrifuged before being added to the cells. The cells were then incubated for 5 min with shaking at 135 rpm. PEI (25kDa linear PEI, Polysciences, Warrington, PA, USA) was resuspended in FEM at 0.5 μg/μL, vortexed, and briefly centrifuged before it was added dropwise to the culture. At 24 h post-transfection, the cells were diluted 1:1 in fresh FEM. The supernatant was collected 72 h post-transfection by centrifugation (10 min, 500× *g*). The supernatant was then purified by immobilized metal-affinity chromatography using 1 mL HisTrap columns (GE Healthcare, Chicago, IL, USA) according to the manufacturer’s instructions, with the protein being eluted in a single elution step with 250 mM imidazole. The protein was concentrated through an Amicon Ultra filter with a 3000 Da MWCO membrane (Millipore, Burlington, MA, USA) at 5000× *g* for 20–30 min and the protein concentration was determined based on A230 measurement using a Nanodrop spectrophotometer. The final protein preparation was stored at −80 °C until use.

### 2.4. Expression and Purification of HBV-CLPs

The *E. coli* Rosetta strain (Novagen, Darmstadt, Germany) was transformed with the various HBV-CLP-encoding plasmids (i.e., pET28a-HVBc-JUNV Loop 3, pET28a-HVBc-JUNV Loop 7, pET28a-HVBc-JUNV Loop 10, or pET28a-HVBc-FLAG). The bacteria were grown in Luria Bertani medium (1% tryptone, 0.5% yeast extract; 171 mM NaCl; pH 7.0) supplemented with 30 μg/mL Kanamycin and 1% glucose at 37 °C until an OD600 of 0.6 was reached. Protein expression was then induced by the addition of 1 mM (final concentration) isopropyl β-d-1-thiogalactopyranoside, with further incubation at 37 °C for 3 h. The bacteria were collected by centrifugation at 8000× *g* for 20 min and the pellet was resuspended in Buffer W (100 mM Tris/HCl pH 8.0, 150 mM NaCl, 1 mM EDTA) supplemented with Proteoloc (Expedeon, Cambridge, UK). The suspension was sonicated on ice 3 times with intermittent pulsation for 1 min and followed by a 1 min pause to allow the sample to cool down. The resulting lysate was centrifuged at 10,000× *g* for 30 min. The supernatant was then loaded onto a Strep-Tactin Sepharose column (IBA Biotech) as previously described [18,19]. The column was pre-equilibrated with Buffer W, washed with 5 column volumes of Buffer W, and the proteins were eluted in Buffer W supplemented with 2.5 mM desthiobiotin (IBA Lifesciences, Göttingen, Germany). The fractions were analyzed using SDS-PAGE followed by staining with Instant blue (Expedeon, Cambridge, UK). Fractions that contained HBV core protein were concentrated using an Amicon Ultra filter with a 10,000 Da MWCO (Millipore, MA, USA) at 5000× *g* for 20–30 min before being analyzed again by SDS-PAGE with staining using Instant blue (Expedeon, Cambridge, UK) and in Western blot (Supplementary Materials Figure S1) with an HRP-conjugated anti-Strep tag antibody (1:30,000; StrepMAB Classic-HRP; IBA Lifesciences, Göttingen, Germany). The protein concentration was estimated by comparison with a gradient of BSA standards. The final CLP preparations were stored at −80 °C until use.

### 2.5. Characterization of HBV-CLPs by Electron Microscopy

The HBV-CLP preparations, generated as described above, were diluted to a concentration between 50 and 100 ng/μL for analysis by electron microscopy. The protein samples were then immobilized on mesh copper grids and negative stained with 2% uranyl acetate, as previously described [18]. Samples were analyzed on a Zeiss TEM EM10 or Joel TEM 1400 electron microscope at 100 kV.

### 2.6. Sequencing of JUNV GP1

RNA was extracted from stocks of the JUNV strain P3551 and strain XJ13 using the QIAamp Viral RNA Mini Kit (Qiagen, Düsseldorf, Germany). The complete GP ORFs were amplified by RT-PCR using primers binding at the 5′ genome terminus and in the NP gene, respectively. Sanger sequencing was performed by Eurofins Genomics and the data were analyzed in Geneious Prime v. 2021.0.1 (Biomatters, Auckland, New Zealand). The resulting consensus sequences were submitted to GenBank under the accession numbers MZ408913 (strain P3551) and MZ408914 (strain XJ13).

### 2.7. ELISA Assay

Microlon medium binding plates (Greiner, Frickenhausen, Germany) were coated overnight at 4 °C with 100 ng of recombinant JUNV GP1-Strep, JUNV GP1-His, or an irrelevant Strep-tagged antigen (i.e., the amino terminal domain of Schmallenberg virus (SBV Gc-Strep; [17])) diluted to 1 ng/µL in carbonate buffer (1.59 g/L Na_2_CO_3_, 2.93 g/L NaHCO_3_, pH 9.6). Heat-inactivated serum samples (56 °C, 30 min) diluted 1:10 in PBS + 0.05% Tween were incubated for 1 h at room temperature, followed by incubation with Protein G-HRP (1:50,000, Merck, Darmstadt, Germany, cat # 539322) diluted in PBS + 0.05% Tween + 2% Fish Skin Gelatin (Sigma-Aldrich, MA, USA) for 45 min at room temperature. Signals were detected using 3,3′,5,5′-tetramethylbenzidine (TMB, AbCam, Cambridge, UK) with incubation in the dark for 3 min, after which 0.5 M H_2_SO_4_ was added, and absorption was measured at 450 nm.

### 2.8. Immunization and Collection of Serum Samples

Adult 10-week-old (approximately 2 kg) New Zealand white rabbits obtained from the breeding facility of the Friedrich-Loeffler-Institut were immunized three times with either JUNV GP1-Strep or HBV-CLPs expressing one of the three JUNV-derived peptide sequences (i.e., Loop 3, Loop 7, or Loop 10) or the control peptide (FLAG). Each immunization consisted of 20 µg of protein in GERBU Adjuvant P (Gerbu Biotech) at each of 4 injection sites (i.e., 80 µg of antigen per animal). Immunizations were spaced 21–42 days apart, with samples collected 14 days after each immunization. Blood was collected under anesthesia from the jugular vein. Serum was prepared from whole blood by allowing the samples to clot at room temperature followed by centrifugation at 1500× *g* for 10 min.

### 2.9. Transcription and Replication-Competent Virus-like Particle (trVLP) Neutralization Assay

JUNV trVLPs were generated as previously described [20,21]. Briefly, BSR-T7/5 cells were transfected with plasmids encoding JUNV NP, JUNV L, a nanoluciferase-expressing JUNV S-segment minigenome, and T7. After 24 h, cells were further transfected with plasmids encoding JUNV Z and either the homologous JUNV (strain Romero) GP or plasmids encoding GPs from different JUNV strains, as indicated (i.e., strain Espindola, P3551, or XJ13). To assess neutralization, trVLP preparations were incubated 1:1 with a two-fold dilution series of heat-inactivated (56 °C, 30 min) serum samples from either human Candid#1 vaccinees or immunized rabbits, as indicated. Samples were incubated for 2 h at 37 °C before 100 µL of the trVLP/serum mixture were applied to Huh7 cells pre-transfected with JUNV NP and JUNV L, as previously described [20]. For each sample, 4 biological replicates per experiment were performed. Reporter activity was measured after 48 h using NanoGlo substrate (Promega, Madison, WI, USA) on a GloMax Discover microplate reader (Promega, WI, USA).

### 2.10. Plaque Reduction Neutralization Assay

JUNV (strain CbaIV4454) stocks were diluted to 1000 pfu/mL and incubated 1:1 with a 2-fold dilution series of the indicated heat-inactivated (56 °C, 30 min) rabbit or human serum samples. Samples were incubated for 1 h at 37 °C before 100 μL of the virus/serum mixture were applied to Vero cells grown in 24-well microplates. Monolayers were then incubated with 1 mL of semi-solid medium (1.4% methylcellulose + 2 × MEM with 3% FCS) and plates were incubated for 7 days before being fixed in 10% formalin and stained with 1% crystal violet.

### 2.11. Statistical Analysis

One-way ANOVA was performed in GraphPad Prism, v. 8 (GraphPad, https://www.graphpad.com/). Post-hoc tests were performed using Sidak’s test (for comparison of selected pairings) or Dunnett’s test (for comparison to a control). The following significance cut-offs were used: * *p* ≤ 0.05; ** *p* ≤ 0.01; *** *p* ≤ 0.001; **** *p* ≤ 0.0001.

## 3. Results

### 3.1. Immunogen Design

Since the goal of this study was to induce a strong neutralizing antibody response that impairs the interaction of JUNV with its receptor, the receptor-binding JUNV GP1 subunit was chosen as the basis for the generated immunogens. Recombinant GP1 and peptide sequences were based on the JUNV strain CbaIV4454. However, examination of the sequences for all 63 naturally occurring JUNV isolates available in GenBank showed that the sequences of these loops are generally well conserved, with only a limited degree of variation at specific positions (Figure 1A and Figure S2). More specifically, with respect to the GP sequences from the viruses/trVLPs used for the analysis of neutralization in this study (i.e., strains CbaIV4454, Romero, Espindola, P3551, and XJ13), variability was observed only at amino acid positions 111 and 116 in Loop 3, and position 168 in Loop 7 (Figure 1B). In particular, the T168A mutation in Loop 7, which occurs among later-stage Candid#1 precursors, and affects a putative glycosylation site [22], was already present in our XJ13 isolate (Figure 1B).

### 3.2. Expression and Purification of Recombinant GP1 and HBV–CLPs Expressing JUNV GP1 Peptide Loops

**Figure 2:**
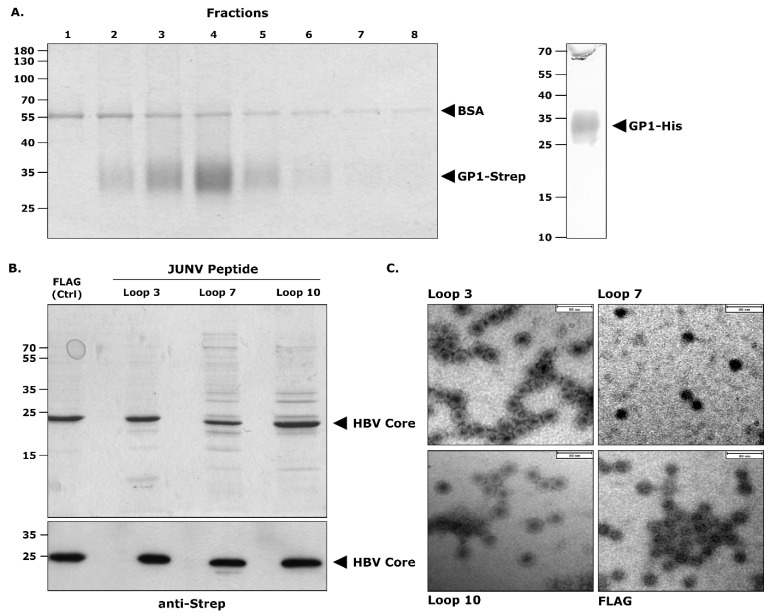
Preparation of antigens for immunization. (**A**) Purification of recombinant Junín virus (JUNV) GP1. JUNV GP1 containing a C-terminal Strep tag (GP1-Strep) was transiently expressed in HEK-293 cells and purified via affinity chromatography using Strep-Tactin resin. The eluted fractions were analyzed by Coomassie staining (left panel). Alternatively, JUNV GP1 containing a C-terminal hexahistidine (GP1-His) tag was expressed in Freestyle 293-F cells and purified via affinity chromatography using HisTrap columns. The resulting protein fraction was also analyzed by Coomassie staining (right panel). (**B**) Hepatitis B virus core-like particles (HBV-CLPs) carrying JUNV GP1 peptide loops. HBV-CLPs containing the indicated antigens (i.e., Loop 3, Loop 7, Loop 10, or a control FLAG tag) were produced in *E. coli* and isolated from the soluble fraction of lysates via Strep tag-mediated affinity purification. Eluates were analyzed by Coomassie staining (upper panel) and Western blot with an anti-Strep antibody (1:30,000; StrepMAB Classic-HRP; IBA Lifesciences, Göttingen, Germany; lower panel). The position of the HBV core protein is indicated. (**C**) Electron microscopic analysis of HBV-CLPs. The purified HBV-CLPs generated in (**B**) were further analyzed by electron microscopy with negative staining. The scale bar shown represents 80 nm.

Recombinant JUNV GP1 (rGP1) was expressed in mammalian cells with either a twin Strep tag (GP1-Strep) or a 6 × His tag (GP1-His) at the C-terminus. Both antigens were purified via affinity chromatography to >90% purity, as assessed by Coomassie staining (Figure 2A). Similarly, HBV-CLPs containing JUNV hTfR1-interacting loops (i.e., Loop 3, Loop 7, and Loop 10) or a FLAG tag at the c/e1 immunodominant epitope could be affinity purified to >80% purity based on Coomassie staining (Figure 2B, upper panel). Further, the bands corresponding to the size of the HBV core protein reacted with a Strep tag-specific antibody, confirming their identity (Figure 2B, lower panel). The particulate nature of the CLP preparations was verified by electron microscopy, which demonstrated the presence of HBV-CLPs approximately 40 nm in diameter (Figure 2C).

### 3.3. Immunization with rGP1 Elicits Antibodies with Neutralizing Activity

**Figure 3:**
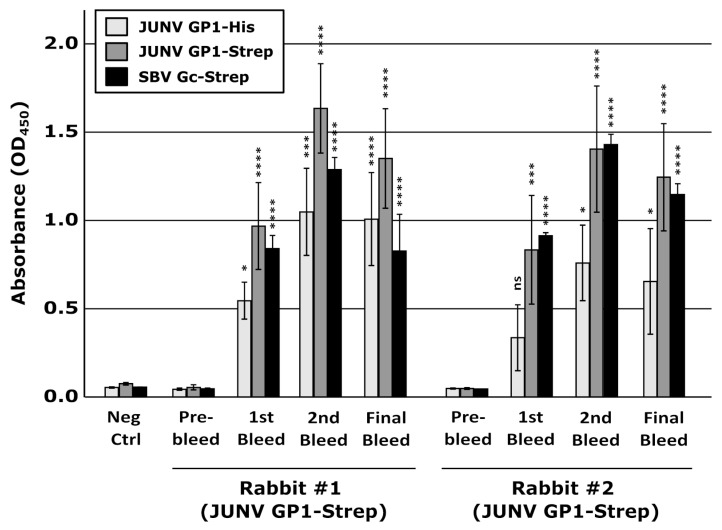
Development of antibodies following vaccination with recombinant Junín virus (JUNV) GP1. Blood samples were collected from rabbits either before immunization or 14 days after each of 3 immunizations with 80 ug of recombinant JUNV GP1-Strep. A sample from another rabbit unrelated to this study served as an additional control (Neg Ctrl). Serum fractions were diluted 1:10 for use in an indirect ELISA assay with JUNV GP1-Strep, JUNV-GP1-His, or Schmallenberg virus Gc amino-Strep (SBV-Gc-Strep) as the target antigen, as indicated. Data from at least two independent experiments are represented and the results of a one-way ANOVA comparing samples post-vaccination to the respective pre-immunization samples are shown (ns not significant; * *p* ≤ 0.05; *** *p* ≤ 0.001; **** *p* ≤ 0.0001).

In order to induce a humoral immune response to JUNV GP1, purified rGP1-Strep was used to immunize two rabbits using a prime/boost/boost approach. The resulting sera were assessed by indirect ELISA for their reactivity against three antigens: rGP1-Strep, rGP1-His, and the unrelated SBV Gc-Strep. A strong immune response was observed against all three antigens upon immunization and remained stable after the first boost, indicating robust antibody production against both the JUNV GP1 and the Strep components of the antigen (Figure 3). The reactivity of the sera against GP1-Strep was also stronger than that against GP1-His, consistent with the detection of this antigen by both the GP1 and Strep-directed antibodies. Further, while this indicates that tags, e.g., a Strep tag, at the C-terminus of JUNV GP1 are highly immunogenic, such a Strep tag-specific reaction did not preclude a strong antibody response against the specific viral antigen, i.e., JUNV rGP1.

**Figure 4:**
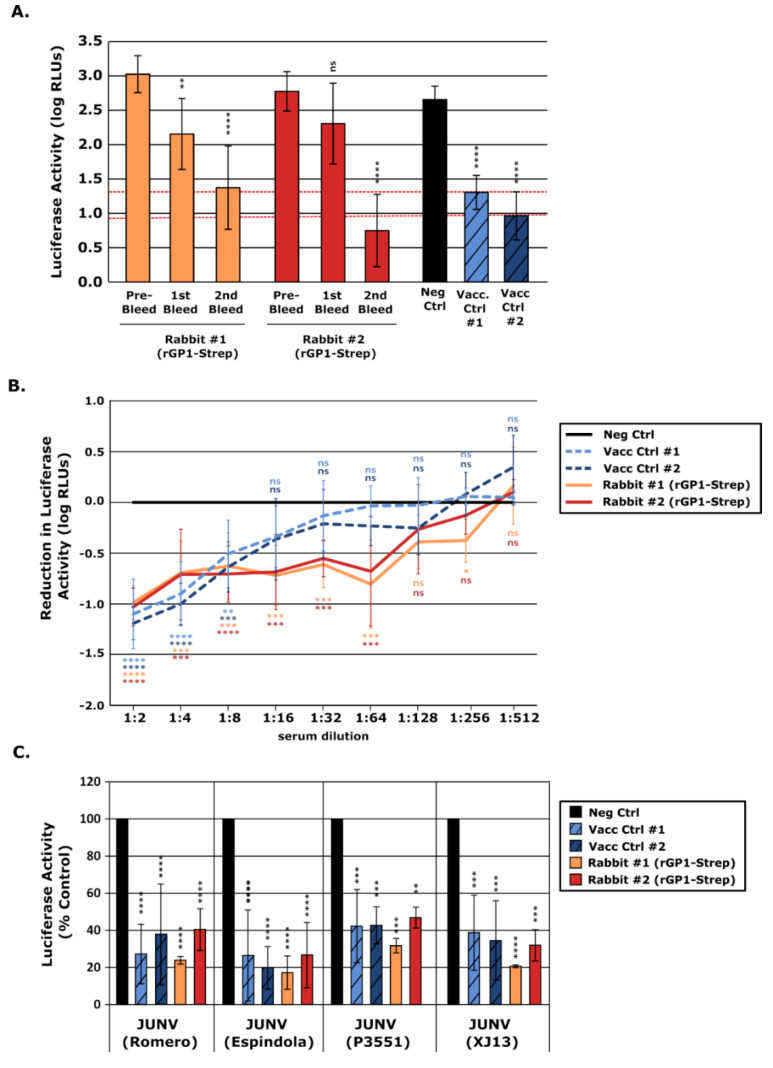
Assessment of neutralizing antibody development following vaccination with recombinant Junín virus (JUNV) GP1 based on neutralization of transcription and replication-competent virus-like particles (trVLPs). (**A**) Development of neutralizing antibodies in response to vaccination. trVLPs containing the GP of JUNV (strain Romero) were incubated 1:1 (i.e., final 1:2 dilution) with serum from rabbits vaccinated with rGP1-Strep collected before or during the immunization process, as indicated. Serum from human Candid#1 vaccinees (Vacc Ctrl) or a control pooled human AB serum served as additional controls. Samples were incubated for 2 h at 37 °C before being used to infect Huh7 cells pre-transfected with plasmids expressing JUNV NP and L. After 48 h, these cells were harvested and their nanoluciferase activity measured (as a measure of viral RNA synthesis). Data from at least two independent experiments are represented and the results of a one-way ANOVA comparison to control antibody treated samples are shown. (**B**) Titration of neutralizing antibodies. Neutralization of trVLPs was performed as in (**A**) using the serum collected after the final immunization. The sera were prepared as a 1:2 dilution series before being added to trVLPs to achieve a dilution range from 1:2 to 1:512. Data from at least three independent experiments are represented and are shown relative to values obtained with the negative control sera. The results of a one-way ANOVA comparing samples to the negative control serum are shown. (**C**) Neutralization of trVLPs expressing JUNV GP from different virus strains. JUNV trVLPs expressing heterologous GP proteins from four different JUNV strains (i.e., Romero, Espindola, P3551, and XJ13) were generated and used in neutralization assays as described in (**A**) with the serum samples collected after the final immunization. Data from at least two independent experiments are represented and the results of a one-way ANOVA to compare samples to a negative control serum are shown (ns not significant; * *p* ≤ 0.05; ** *p* ≤ 0.01; *** *p* ≤ 0.001; **** *p* ≤ 0.0001).

In light of the robust antibody responses in ELISA, we further evaluated the neutralizing activity of the antibodies raised against rGP1-Strep. This was first done using trVLPs to allow testing under BSL-1/2 conditions (i.e., without the need for infectious virus), and also to allow the use of GPs from different JUNV strains to assess the breadth of neutralization [21]. When used undiluted, sera generated against rGP1 already showed evidence of significant levels of neutralization for one animal following the priming immunization (i.e., after the first bleed), which then increased to become significant for both animals following additional immunization (i.e., after the second bleed) (Figure 4A). Titration of the serum obtained after the second boost (i.e., after the third/final bleed) (Figure 4B) showed neutralizing antibody levels that were sufficient to produce statistically significant reductions in the reporter activity out to a 1:64 dilution. These levels were higher than for positive control samples from long-term human vaccinees (sera collected 9 years post-vaccination), which were only significant up to a dilution of 1:8. Further, similar to human Candid#1 vaccinees, the rGP1 rabbit sera showed efficient neutralization of trVLPs containing the GPs of 4 different unrelated JUNV strains, i.e., Romero, Espindola, P3551, and XJ13 (Figure 4C).

**Figure 5:**
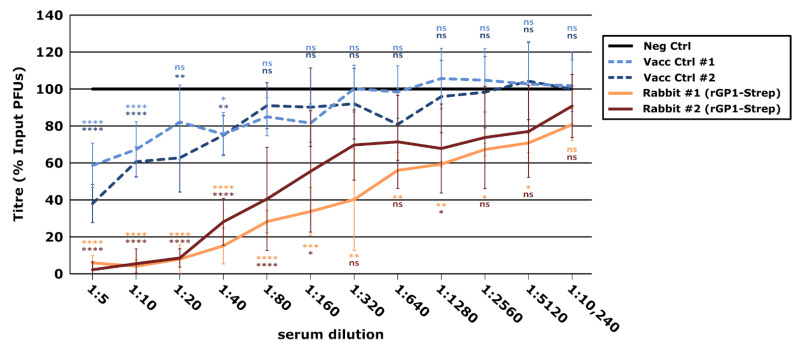
Assessment of neutralizing antibody development following vaccination with recombinant Junín virus (JUNV) GP1 based on the plaque reduction neutralization test. JUNV (strain Cba IV4454) diluted to 10^3^ pfu/mL was mixed 1:1 with serum from rabbits vaccinated with recombinant JUNV GP1-Strep or from a naïve rabbit. Serum from human Candid#1 vaccinees (Vacc Ctrl) or a control pooled human AB serum served as additional controls. Samples were incubated for 1 h at 37 °C before 100 μL were used to infect Vero cells for analysis using plaque assay. Values are expressed as a percentage of the number of plaques in samples treated with the control serum (i.e., naïve rabbit or human AB serum). Data from at least two independent experiments are represented and the results of a one-way ANOVA comparison of experimental samples with the control antibody are shown (ns not significant; * *p* ≤ 0.05; ** *p* ≤ 0.01; *** *p* ≤ 0.001; **** *p* ≤ 0.0001).

Finally, we tested the neutralizing activity of the rabbit sera raised against rGP1-Strep using the plaque reduction neutralization test (PRNT), which is the gold standard for assessing JUNV neutralization [23]. Importantly, these assays also used the same JUNV strain (i.e., CbaIV4454) upon which the rGP1 and Loop peptide sequences were based. The sera also exhibited high levels of JUNV neutralization, reducing input virus infection by 90% at dilution values of 1:20. The calculated PRNT_50_ values were 1:640 and 1:160 for rabbits #1 and #2, respectively (Figure 5). Moreover, both sera showed greater neutralization than the positive control long-term human vaccinee sera, which had PRNT_50_ values of 1:40 or less, and where a 90% reduction in virus infection could not be achieved even at the lowest assayed dilution. Overall, the data from these experiments clearly indicate that immunization with rGP1 alone is sufficient to induce a strong neutralizing antibody response against JUNV GP.

### 3.4. HBV-CLPs Expressing Receptor-Interacting Loops of JUNV GP1 Elicit Antibodies, but These Do Not Have Neutralizing Activity

**Figure 6:**
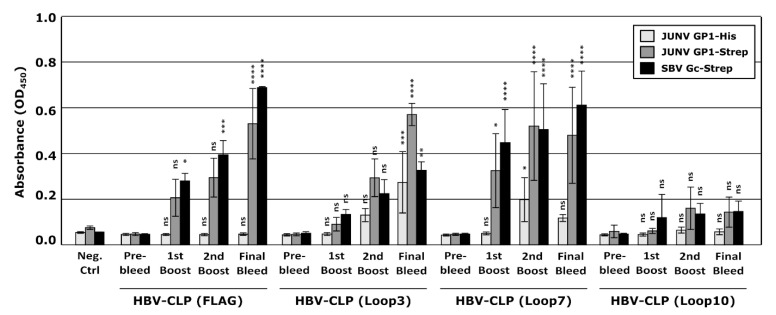
Development of antibodies following vaccination with Hepatitis B virus core-like particles (HBV-CLPs) expressing Junín virus (JUNV)-derived peptides. Blood samples were collected from rabbits either before immunization or 14 days after each of 3 immunizations with 80 µg of purified HBV-CLPs expressing 1 of 3 JUNV-derived peptides (i.e., Loop 3, Loop 7, Loop 10), as indicated. Vaccination with HBV-CLPs without a JUNV-specific antigen (i.e., HBV-CLP (FLAG)) and a sample from another rabbit unrelated to this study (Neg Ctrl) served as additional controls. Serum fractions isolated from these samples were diluted 1:10 for use in an indirect ELISA assay with JUNV GP1-Strep, JUNV-GP1-His, or Schmallenberg virus Gc amino-Strep (SBV-Gc-Strep) as the target antigen, as indicated. Data from at least two independent experiments are represented and the results of a one-way ANOVA comparing post-vaccination samples to the respective pre-bleed samples are shown (ns not significant; * *p* ≤ 0.05; ** *p* ≤ 0.01; *** *p* ≤ 0.001; **** *p* ≤ 0.0001).

To examine the contribution of specific JUNV GP1 epitopes in eliciting neutralizing antibodies, the individual GP1 peptide loops that interact with TfR1 were expressed on the surface of HBV-CLPs and used to immunize rabbits, again using a prime/boost/boost approach. Animals immunized with HBV-CLP-FLAG (control), Loop 3, or Loop 7 all produced strong antibody responses against the GP1-Strep and SBV-Gc-Strep in ELISA. However, while the HBV-CLP-FLAG sera showed the expected lack of response towards GP1-His, Loop 3 and Loop 7-directed sera also produced very modest responses to the GP1-His protein (Figure 6), indicating that HBV-CLPs mainly stimulated anti-Strep tag antibodies. The rabbit immunized with HBV-CLP-Loop 10 did not mount a strong immune response against any of the three antigens tested, indicating a lack of both Strep- and JUNV-directed antibodies.

**Figure 7:**
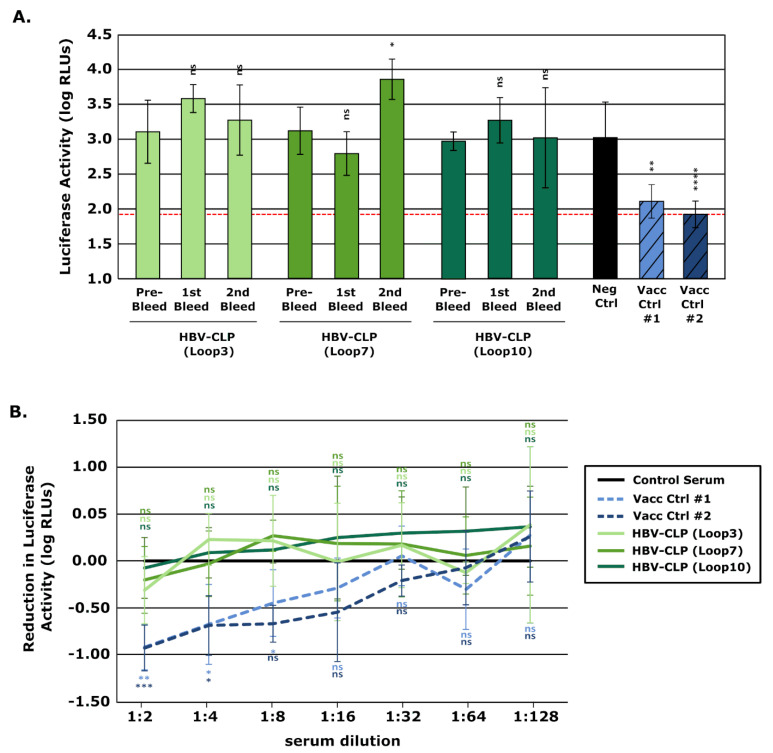
Assessment of neutralizing antibody development following vaccination with Hepatitis B virus core-like particles (HBV-CLPs) expressing Junín virus (JUNV)-derived peptides using neutralization of transcription and replication-competent virus-like particles (trVLPs). trVLPs containing the GP of JUNV (strain Romero) were incubated 1:1 (i.e., final 1:2 dilution) with serum from rabbits vaccinated with HBV-CLPs expressing 1 of 3 JUNV-derived peptides (i.e., Loop 3, Loop 7, Loop 10) collected before or during the immunization process, as indicated. Serum from human Candid#1 vaccinees (Vacc Ctrl) or a control pooled human AB serum served as additional controls. Samples were incubated for 2 h at 37 °C before being used to infect Huh7 cells pre-transfected with plasmids expressing JUNV NP and L. After 48 h, these cells were harvested and their nanoluciferase activity measured (as a measure of viral RNA synthesis). Data from at least two independent experiments are represented and the results of a one-way ANOVA comparison with control antibody-treated samples are shown. (**B**) Titration of neutralizing antibodies. Neutralization of trVLPs was performed as in (**A**) using the serum collected after the final immunization. The sera were prepared as a 1:2 dilution series before being added to trVLPs to achieve a dilution range from 1:2 to 1:128. Data from four independent experiments are represented and are shown relative to values obtained with the negative control sera (i.e., HBV-CLP-FLAG or human AB serum). The results of a one-way ANOVA comparing samples to the negative control serum are shown (ns not significant; * *p* ≤ 0.05; ** *p* ≤ 0.01; *** *p* ≤ 0.001; **** *p* ≤ 0.0001).

**Figure 8:**
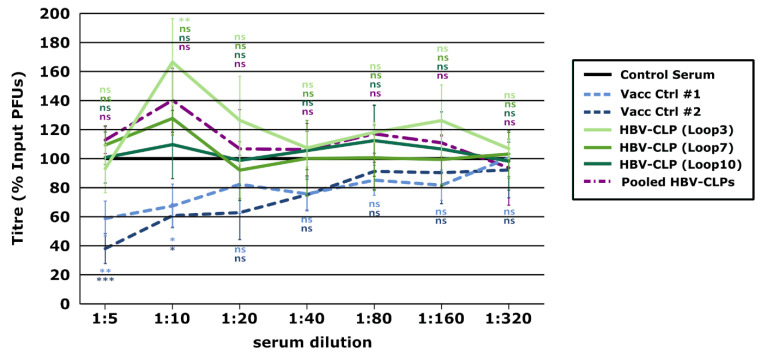
Assessment of neutralizing antibody development following vaccination with hepatitis B virus core-like particles (HBV-CLPs) expressing Junín virus (JUNV)-derived peptides based on the plaque reduction neutralization test. JUNV (strain Cba IV4454) diluted to 10^3^ pfu/mL was mixed 1:1 with serum from rabbits vaccinated with 1 of the HBV-CLPs indicated (Loop 3, Loop 7, or Loop 10), a pooled mixture of the sera from these rabbits, or serum from a rabbit that received a control HBV-CLP containing a FLAG peptide. Serum from human Candid#1 vaccinees (Vacc Ctrl) or a control pooled human AB serum served as additional controls. Samples were incubated for 1 h at 37 °C before 100 μL were used to infect Vero cells for analysis using the plaque assay. Values are expressed as a percentage of the number of plaques in samples treated with the control serum (i.e., HBV-CLP-FLAG or human AB serum). Data from at least two independent experiments are represented and the results of a one-way ANOVA comparison of the experimental samples with the control antibody are shown (ns not significant; * *p* ≤ 0.05; ** *p* ≤ 0.01; *** *p* ≤ 0.001).

Analysis of the neutralizing activity of these Loop-directed sera using trVLPs showed no reduction in reporter signals following incubation with any of the sera collected during the immunizations even when used undiluted (Figure 7A,B), indicating the absence of even a weak neutralizing anti-GP1 immune response. The plaque reduction neutralization assay also confirmed that immunization with HBV-CLPs expressing the different JUNV GP1-derived loops did not elicit virus-neutralizing antibodies even at the lowest dilutions used (i.e., 1:5) (Figure 8). Additionally, even when the sera against the different HBV-CLPs were combined and tested as a pool, no synergistic effect was observed. The data, therefore, suggest that these JUNV GP1 loops alone are insufficient to induce neutralizing antibodies that react with GP, even when presented using HBV-CLPs as a scaffold.

## 4. Discussion

The effectiveness of plasma therapy in both humans [24] and animal models [25], and the recent success using a humanized monoclonal antibody in JUNV-infected nonhuman primates [26] clearly indicate that antibody-mediated virus neutralization plays an important role in protecting against AHF. The arenavirus GPC also effectively mediates protection when used as an immunogen in various vaccine platforms [25,27], while immunization of mice [28] and horses [29] with recombinant JUNV GP1 alone results in a strong neutralizing antibody response that blocks the entry of pseudotypes bearing the JUNV GPC. Building on this evidence suggesting both therapeutic and prophylactic utility of GP1-elicited immune responses, we assessed the potential of both JUNV GP1 and individual epitopes from the receptor-binding domain to elicit antibodies with neutralizing activity.

In examining the immunogenicity of JUNV GP1, we focused on mammalian-expressed recombinant protein containing a Strep tag. Importantly, while such tags are frequently included to facilitate purification and detection of the antigen during production, as we have done here, it is necessary to consider their impact on antigen conformation and immunogenicity. For instance, the presence of a hexahistine tag in the *Streptococcus pneumoniae* SP0845 protein has been shown to lead to oligomerization and a higher β-sheet content compared to the untagged protein [30]. Further, while tags can be designed so that they can be removed post-purification, through protease cleavage, this adds to the costs and workflow complexity of antigen production, and it is not guaranteed that removal of the tag would facilitate refolding of a protein whose conformation is affected by its presence during expression. Importantly, however, the sera raised in rabbits against JUNV GP1-Strep in this study reacted strongly to both the homologous GP1-Strep and GP1 with an unrelated tag (GP1-His) in ELISA. Unsurprisingly, detection was consistently stronger with GP1-Strep, indicating a contribution of anti-Strep tag antibodies as well, which was also confirmed by the detection of an unrelated Strep-tagged protein (SBV Gc-Strep) by these sera. Nonetheless, there is clearly also strong reactivity towards the JUNV GP1 portion of the antigen, indicating that the use of a Strep tag for antigen purification does not pose a barrier to the purification of NW arenavirus GP1 that remains highly immunogenic. Correspondingly, we also found that immunization with GP1-Strep induced a strong virus-neutralizing immune response in rabbits, as shown by PRNT, and confirming the reactivity of the generated antibodies with native antigen, including when it is presented in the context of mature GP on intact viral particles. Taken together with another recent study that has shown that rabbits immunized with VLPs carrying the entire glycoprotein of the OW arenavirus Lassa virus also develop high titers of broadly virus-neutralizing antibodies [31], our results thus support a general applicability of rabbits for the production of neutralizing antibodies against both OW and NW arenaviruses.

Interestingly, while monoclonal antibodies generated against JUNV have so far all been either murine [32,33,34] or human [35], it has recently been shown that rabbit-derived monoclonal antibodies (mAbs) against the SARS-CoV2 spike protein recognize novel epitopes not recognized by known human or murine mAbs [36]. This suggests that the isolation of JUNV GP1-specific rabbit mAbs not only has the potential to lead to the discovery of antibodies that could be developed as immunotherapeutics upon humanization, but that these may target different epitopes and/or exhibit different and/or complementary activities compared to those produced using other approaches [37]. Indeed, the need for complementary antibody activities (i.e., neutralization and antibody-dependent cell cytotoxicity) has recently been shown to be critical for successful monoclonal antibody-based treatment of Ebola virus (reviewed in [38]). To this end, the recombinant JUNV GP1 prepared in this study would also be a suitable reagent for the sorting of Ig+ and GP1+ B-cells via its C-terminal Twin Strep tag. Our results also provide support that JUNV GP1 is a relevant antigen for subunit vaccine development. While this has also been suggested by experiments with immunized mice [28] and horses [29], our data provide evidence that this is also the case in rabbits, and thereby extends the generalizability of this observation to additional species. Given this increasing amount of data now reporting successful JUNV GP1-based immunization [28,29], it appears that the generation of a multivalent vaccine against pathogenic NW arenaviruses based on GP1 may be feasible. In particular, the use of covalently linked glycoprotein domains, which could be readily produced using an approach such as we describe here for JUNV GP1, represents a promising option. A similar approach, based on the glycoprotein domains from two different orthobunyaviruses, has recently been shown to stimulate the production of neutralizing antibodies against both viruses [39], suggesting that an antigen comprising linked copies of the GP1 of JUNV, MACV, and Guanarito virus could potentially provide protection against all three pathogens. Alternatively, the GP1 subunits of multiple NW arenaviruses could be coupled to a scaffold using a “plug-and-display” system [40], such as that recently used to induce a protective immune response against MERS-CoV [41].

While antibody binding to sites unrelated to receptor recognition can also contribute to neutralization (for instance, by preventing conformational rearrangement within the glycoprotein), for NW arenaviruses there is clear evidence that the GP1–TfR1 interface is both immunogenic, and that blocking it efficiently inhibits infection. Specifically, mAbs binding to either the receptor-interacting regions of JUNV GP1 [12,33,35] or the virus-interacting apical domain on TfR1 [9,42] effectively neutralizes infection. Further, some mAbs (e.g., CR107) are also able to cross-neutralize MACV by targeting a conserved epitope within the receptor-binding domain [35]. Moreover, a recently designed TfR1 mimetic molecule that interacts with the receptor-binding surface of GP1 was also shown to inhibit NW arenavirus infection [43]. However, until now, approaches to specifically target this interaction on the side of the virus, i.e., using the epitopes in GP1 that interact with hTfR1 as an antigen, have not been considered. A major challenge with such approaches is that the short length of the epitopes involved often results in poor immunogenicity if free peptides are used for immunization [44]. One potential solution to increase the immunogenicity of certain antigens is the use of particulate scaffolds for peptide presentation [45,46]. In several instances, these approaches have been shown to induce a strong immune response that in certain cases can also overcome immune tolerance [47,48]. In particular, HBV CLPs can be produced in a cost-effective manner in both bacteria and yeast [49] and assemble into icosahedral structures of either 180 or 240 subunits [50]. Further, their structural plasticity allows the accommodation of foreign antigens within the immunodominant region [16,51], which can lead to both B cell and T cell-mediated immune responses against the targeted pathogen (reviewed in [52]). For instance, immunization with Strep-tagged HBV CLPs encoding two surface antigens of *Neisseria meningitidis*, FHbp and NadA, was recently shown to elicit antibodies against both antigens, with an anti-NadA serum also showing bactericidal activity [30]. Therefore, the suitability of HBV-CLPs to act as a scaffold to present specific epitopes of JUNV GP1, and thereby focus the immune response on those peptides that interact with TfR1, was also evaluated. However, this approach was not successful in stimulating either robust increases in total anti-JUNV GP1 antibody or neutralizing antibody. Nonetheless, the modest reactivity we did observe towards GP1-His by sera produced by immunization with HBV-CLP (Loop 3) and to a lesser extent HBV-CLP (Loop 7) (Figure 6) indicates some limited development of JUNV-specific antibodies against these peptides. Importantly, this suggests that, also in this platform, the presence of the Strep tag does not necessarily preclude the production of antibodies against other antigens, including these small peptide loops, when displayed on HBV-CLPs. In contrast, the strong response seen against the Strep tag in these animals may be due to the presence of two tandem copies of the Strep tag and increased accessibility of the epitope provided by the linker, which may increase its immunogenicity [53,54]. Consequently, it appears likely that, when presented in isolation on the CLPs, these epitopes lack their authentic conformation, and hence do not elicit antibodies reactive with the native antigen. Such conformational differences due to the absence of the appropriate structural context provided by the full-length protein represent a well-known, but difficult to resolve, challenge to peptide-based antibody development [44]. Indeed, close examination of the structure of the JUNV GP1–TfR1 interface (Figure 1) appears to suggest that the conformation of Loops 7 and 10 may be heavily dependent on other interactions within JUNV GP1, although this appears to be less relevant for Loop 3. On the other hand, the poor immunogenicity of these individual sequences could also account for these results. Thus, a possible approach to deal with these issues might be to insert longer peptides from JUNV GP1 within this region, which might then be more immunogenic and/or more likely to adopt an authentic conformation. Finally, the presence of a glycosylation site within Loop 7 potentially poses an additional challenge regarding the antigen expression strategy, as this modification would not be present in bacterially expressed CLPs, such as those used in our study, which might then also affect antigenicity. However, given that glycosylation at this position is variable among JUNV strains (i.e., strains H8027, HLye63, An17246, An5185, and An_8640 all lack this site; Figure S2), a lack of modification at this site is something that an effective antibody response against this region would need to be able to tolerate in order to provide adequate protection against the existing virus diversity found in nature. Nonetheless, future approaches could include the generation of CLPs in systems that allow this post-translational modification, although Loop 7 might then still need to be used in conjunction with additional antigens to ensure reactivity against all JUNV strains.

## 5. Conclusions

Taken together, our results support the potential of JUNV GP1 to induce robust antibody responses, including the generation of neutralizing antibodies, making it a promising target for the development of both subunit vaccines and immunotherapeutics. Further, we report initial efforts to raise antibodies to specific GP1 peptides that interact with the viral receptor TfR1, and while these were so far met with little success, they provide important insights that can inform future approaches. Thus, these results will help support the development of future alternatives for the treatment and prevention of NW arenavirus infections.

## Data Availability

Sequences obtained for the glycoprotein genes of JUNV strain P3551 and strain XJ13 were submitted to GenBank at [https://www.ncbi.nlm.nih.gov/genbank/] under accession numbers MZ408913 and MZ408914, respectively.

## Supplementary Materials

The following are available online at https://www.mdpi.com/article/10.3390/vaccines10020173/s1. Figure S1: Western Blot. Figure S2: Alignment of Junín virus (JUNV) sequences. The sequences of 63 unique naturally occurring JUNV isolates available in GenBank were aligned and trimmed to show only amino acids in and around the peptide loops used for immunization in this study. The location of the sequences against which peptides were generated are shown in color (Loop 3, red; Loop 7, blue; Loop 10, magenta) above the Cba IV4454 reference sequence. Variable amino acids are highlighted in color in each sequence. Text S1. Sequences for expression plasmids used in this study.
